# YF17D-vectored Ebola vaccine candidate protects mice against lethal surrogate Ebola and yellow fever virus challenge

**DOI:** 10.1038/s41541-023-00699-7

**Published:** 2023-07-11

**Authors:** Viktor Lemmens, Lara Kelchtermans, Sarah Debaveye, Winston Chiu, Thomas Vercruysse, Ji Ma, Hendrik Jan Thibaut, Johan Neyts, Lorena Sanchez-Felipe, Kai Dallmeier

**Affiliations:** 1 KU Leuven Department of Microbiology, Immunology and Transplantation, Rega Institute, Virology and Chemotherapy, Molecular Vaccinology & Vaccine Discovery, BE-3000 Leuven, Belgium; 2 KU Leuven Department of Microbiology, Immunology and Transplantation, Rega Institute, Translational Platform Virology and Chemotherapy (TPVC), BE-3000 Leuven, Belgium; 3 GVN, Global Virus Network, Baltimore, MD USA; 4 Present Address: AstriVax, BE-3001 Heverlee, Belgium

**Keywords:** Live attenuated vaccines, Viral infection

## Abstract

Ebola virus (EBOV) and related filoviruses such as Sudan virus (SUDV) threaten global public health. Effective filovirus vaccines are available only for EBOV, yet restricted to emergency use considering a high reactogenicity and demanding logistics. Here we present YF-EBO, a live YF17D-vectored dual-target vaccine candidate expressing EBOV glycoprotein (GP) as protective antigen. Safety of YF-EBO in mice was further improved over that of parental YF17D vaccine. A single dose of YF-EBO was sufficient to induce high levels of EBOV GP-specific antibodies and cellular immune responses, that protected against lethal infection using EBOV GP-pseudotyped recombinant vesicular stomatitis virus (rVSV-EBOV) in interferon-deficient (*Ifnar*^-/-^) mice as surrogate challenge model. Concomitantly induced yellow fever virus (YFV)-specific immunity protected *Ifnar*^-/-^ mice against intracranial YFV challenge. YF-EBO could thus help to simultaneously combat both EBOV and YFV epidemics. Finally, we demonstrate how to target other highly pathogenic filoviruses such as SUDV at the root of the 2022 outbreak in Uganda.

## Introduction

Ebola virus (EBOV), a member of the *Filoviridae* family, is causing a severe and acute systemic disease in humans known as Ebola virus disease (EVD) with mortality rates up to 80%^1^. Whereas after its discovery in 1976, only sporadic local outbreaks were reported on the African continent, more recently EVD is on the rise sparking large epidemics in West Africa (2013–2016) and the Democratic republic of Congo (DRC) (2018–2020, and 2021), deteriorating societies, economies and political systems in already unstable regions.

The devastating 2013–2016 West African epidemic—with more than 28,000 confirmed cases, 11,000 deaths and an estimated economic cost of over USD 50 billion^2^—fostered an accelerated development of EBOV vaccines to at least protect frontline healthcare workers, of which two have been granted conditional licensure by the European Medicines Agency. Ervebo® (rVSV-EBOV) that is based on replication-competent recombinant vesicular stomatitis virus (VSV) expressing the EBOV glycoprotein (GP) proved effective in an outbreak response^3,4^. However, its high reactogenicity with fever, arthralgia, fatigue and viral dissemination in joints and skin^5–7^, next to a strict requirement for ultradeep cooling during storage and transport precludes its wider use in routine immunization. Likewise, for Zabdeno/Mvabea® (Ad26-ZEBOV/MVA-BN-Filo) a lower estimated efficacy^8^ and an extended 3-month prime-boost regimen are disadvantages limiting its use in the field^9^. Hence potent, safe and convenient second-generation vaccines need to be developed to protect risk populations in EBOV-endemic regions^10,11^.

Yellow fever virus (YFV) is a mosquito-borne flavivirus causing severe hemorrhagic disease in humans. Yellow fever (YF) is endemic in Central and South America, as well as sub-Saharan Africa, where EVD surges. Despite the availability of a very efficient live-attenuated yellow fever vaccine (YF17D), annually an estimated 51,000–380,000 severe cases of YF still occur resulting in 19,000–180,000 deaths^12^. Re-emergence of YF outbreaks can be mainly attributed to a low vaccine coverage due to supply issues of the vaccine^13^. Therefore, alike for EVD, second-generation YFV vaccines with a sustainable supply are needed^14,15^.

YF17D is considered one of the most effective vaccines, stimulating a broad range of innate immune responses resulting in strong humoral and polyfunctional cellular immune responses, which can provide long lasting protection after a single dose^16^. Due to its excellent immunogenic properties, YF17D has been successfully used as a vector platform for the development of novel vaccines^17^.

Since YFV and EBOV share their endemicity, a single vaccine that could simultaneously contribute to the elimination of yellow fever epidemics and combat surges of EVD would be of great benefit in routine immunization programs in endemic regions. Here we report on the development of such a dual-target YF17D-vectored Ebola vaccine candidate (YF-EBO) expressing EBOV GP from a replication-competent full-length YF17D backbone. A single shot of YF-EBO induces both EBOV- and YFV-specific humoral and cellular immune responses in mice. This translated into dual protection from both stringent surrogate EBOV and YFV challenge in IFN type I receptor-knockout (*Ifnar*^-/-^) mice.

## Results

### Generation and characterization of YF-EBO

**Fig. 1:**
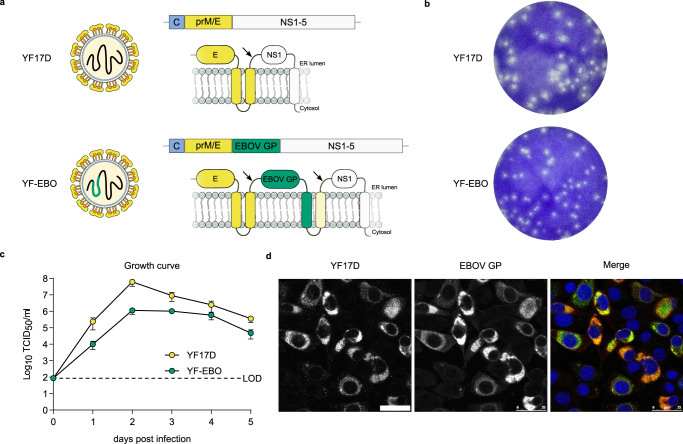
vaccine design and in vitro characteristics. **a** Schematic of YF17D and YF17D-vectored Ebola vaccine candidate (YF-EBO). EBOV GP was inserted into the E/NS1 intergenic region as translational fusion within the YF17D polyprotein inserted in the endoplasmic reticulum (gray). To cope with topological constraints of the fold of both EBOV GP antigen and the polyprotein of the YF17D vector, one extra transmembrane domain (derived from the West Nile virus E protein; light yellow) was added to the C-terminal cytoplasmic domain of the full-length EBOV GP protein. Arrows indicate protease cleavage sites. **b** Representative images of plaque phenotypes from YF17D and YF-EBO on BHK-21J cells, fixed 6 days post-infection. **c** Growth kinetics of YF17D and YF-EBO. BHK-21J cells were infected at a multiplicity of infection (MOI) of 0.01 and virus yields were quantified over time by virus titration on BHK-21J cells. Error bars indicate SEM (*n* = 5) and dashed line represents limit of detection (LOD). **d** Antigenicity of YF-EBO: confocal immunofluorescent images of BHK-21J cells 2 days post-infection with YF-EBO, staining for YF17D (green) and EBOV GP antigen (red) (nuclei stained with DAPI, blue). Scale bar, 25 μm.

YF-EBO was generated by inserting the sequence of the EBOV (Makona strain) glycoprotein (EBOV GP) as translational in-frame fusion at the E/NS1 intergenic region of an infectious cDNA clone of YF17D^17,18^ (Fig. 1a). The YF-EBO vaccine construct was rescued by transfection into BHK-21J cells, which yielded infectious viral progeny. Consistent with the replicative trade-off posed by the insertion of foreign genes into the YF17D backbone, YF-EBO had a smaller plaque phenotype (Fig. 1b) and showed somewhat reduced viral growth kinetics on BHK-21J cells as compared to parental YF17D (Fig. 1c). Expression of both YF17D and EBOV GP antigens was confirmed by immunofluorescent staining of YF-EBO-infected BHK-21J cells with polyclonal YF17D antiserum and an EBOV GP specific antibody (Fig. 1d). To determine the genetic stability of the recombinant vaccine construct, RT-PCR fingerprinting (Supplementary Fig 1A–C) was used to investigate the inserted antigen sequence during serial passages (P2–10) of YF-EBO on BHK-21J cells. The full-length EBOV GP sequence (1932 bp) remained readily detectable until P8 and unchanged as confirmed by direct Sanger sequencing. Only at P10 virus variants with a deletion of 1704 nucleotides (nt) in EBOV GP (between first 57 nt at its 5’end and last 171 nt at its 3’end) started to become dominant. Likewise, proper co-expression of both YF17D and EBOV GP antigens was confirmed by immunofluorescent staining and quantitative high-content imaging of cells that were infected with serially passaged YF-EBO virus (P1–10), whereby EBOV GP expression remained stable for at least eight passages (Supplementary Fig 1D).

**Fig. 2:**
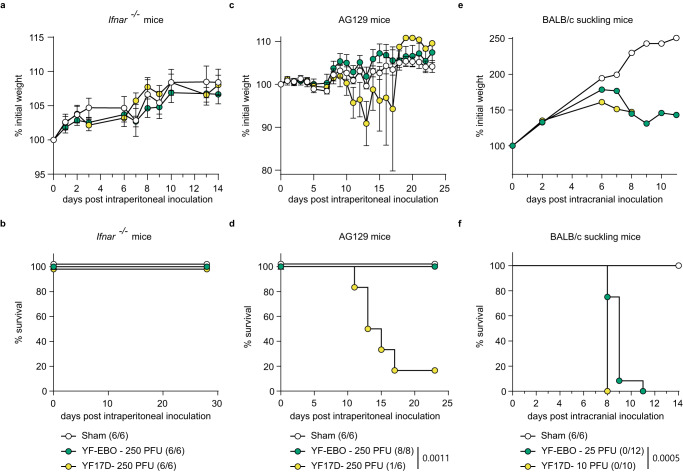
Attenuation of YF-EBO. **a** Weight evolution and **b** survival curve of *Ifnar*^-/-^ mice after intraperitoneal inoculation with 250 PFU of YF-EBO (*n* = 6, green circles), YF17D (*n* = 6, yellow circles), or sham (*n* = 6, white circles) as a control. **c** Weight evolution and **d** survival curve of AG129 mice after intraperitoneal inoculation with 250 PFU of YF-EBO (*n* = 8), YF17D (*n* = 6) or sham (*n* = 6) as a control. **e** Weight evolution and **f** survival curve of 5-day-old suckling BALB/c mice after intracranial inoculation with 25 PFU of YF-EBO (*n* = 12), 10 PFU of YF17D (*n* = 10) or sham (*n* = 6) as a control. The number of surviving mice at study endpoint are indicated within parentheses in the legends and a log-rank test was applied to compare YF-EBO- with YF17D-vaccinated mice, significant *p* values < 0.05 are indicated (**b**, **d**, **f**). Error bars indicate SEM (**a**, **c**).

The safety and attenuation of YF-EBO was compared to YF17D using different mouse models (Fig. 2). First, intraperitoneal inoculation of YF-EBO into 6–8-week-old IFN type I receptor-knockout mice (*Ifnar*^-/-^) was well tolerated, as concluded from a normal weight gain over time (Fig. 2a). To evaluate the neuroinvasive properties of YF-EBO we used IFN type I and II receptor-knockout mice (AG129) which are highly susceptible to neuroinvasive YF17D infection^18^. Intraperitoneal inoculation of 250 plaque forming units (PFU) of YF-EBO (*n* = 8) did not result in any disease symptoms in AG129 mice, whereas a similar dose of YF17D induced neurological symptoms (e.g., paresis, paralysis or a hunched back)^19^, leading to lethality in the vast majority of mice (*n* = 5 of 6) (Fig. 2d). Finally, a neurovirulence test resembling a classical YF17D neuropotency test in BALB/c pups was performed by inoculating 5-day-old pups intracranially with 25 PFU of YF-EBO (*n* = 12) or 10 PFU of YF17D (*n* = 10). Even when using this higher dose of YF-EBO, a slight improvement regarding survival was observed compared to YF17D inoculation (Fig. 2f) (median time to humane endpoint of 9 days, interquartile range (IQR) of 8.25–9 versus 8 days, IQR 8–8, respectively; *p* < 0.0001, log-rank-test).

### EBOV-specific immunogenicity and protection against lethal rVSV-EBOV infection in mice

We used rVSV-EBOV, a replication-competent recombinant VSV pseudotyped with EBOV GP resembling commercial Ervebo®, as a surrogate for EBOV infection in *Ifnar*^-/-^ mice. Likewise, in contrast to wild-type mice that restrict YF17D replication, *Ifnar*^-/-^ mice can readily be immunized using YF17D and are hence a suitable model to study YF17D and YF17D-vectored vaccines^20^. rVSV-EBOV has previously been shown to cause lethal infection in *Stat2*^-/-^or *Ifnar*^-/-^ mice^21,22^, which both lack a functional antiviral interferon (IFN) type I response. Importantly, rVSV-EBOV is a biosafety level (BSL)−2 agent, thus not requiring BSL-4 containment as wild-type EBOV.

To establish our rVSV-EBOV challenge model, *Ifnar*^-/-^ mice were infected intraperitoneally with either 100 (*n* = 3) or 100.000 (*n* = 3) plaque forming units (PFU) of rVSV-EBOV and monitored for the development of disease. A rapid onset of disease symptoms such as weight loss (Supplementary Fig. 2a), inactivity, ruffled fur, hunched posture and lethargy was observed for both doses, resulting in the development of high disease scores (Supplementary Fig. 2b) ultimately requiring euthanasia (Supplementary Fig. 2c). Organs (liver, spleen, kidney, brain and lung) were collected at the day of euthanasia to determine infectious viral loads and rVSV-EBOV replication was found in all collected organs (Supplementary Fig. 2d). The highest infectious viral loads, up to 10^8^ TCID_50_ /30 mg organ, were detected in liver and spleen. Thus, similar to previous studies^21,22^, we observed that intraperitoneal inoculation of only 100 PFU of rVSV-EBOV in *Ifnar*^-/-^ mice results in a systemic lethal infection with rapid onset of disease symptoms. *Ifnar*^-/-^ mice were used in the following to assess vaccine efficacy of YF-EBO.

**Fig. 3:**
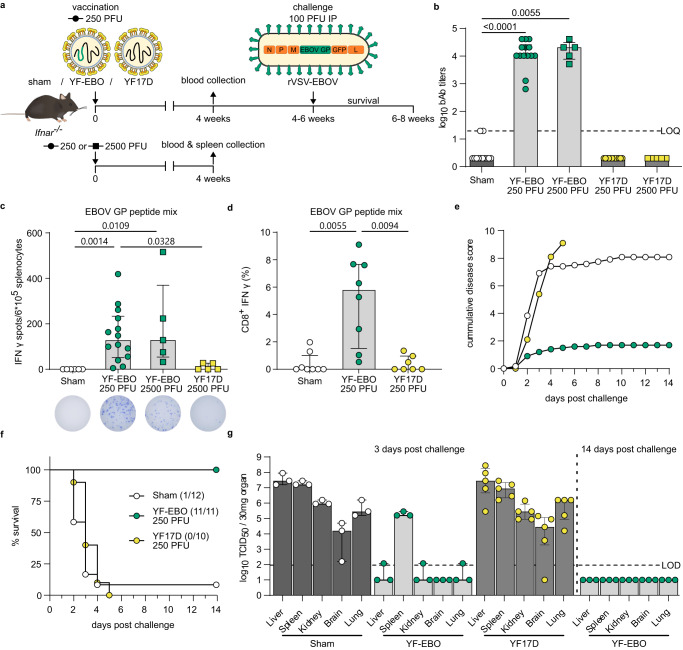
EBOV-specific immunity and protection in mice. **a** *Ifnar*^-/-^ mice were vaccinated intraperitoneally with 250 (circles) or 2500 PFU (squares) of either YF-EBO, YF17D or sham. A subset of mice vaccinated with 250 PFU and all mice vaccinated with 2500 PFU were sacrificed 4 weeks post-vaccination for spleen collection. Remaining mice vaccinated with 250 PFU were challenged intraperitoneally with 100 PFU of rVSV-EBOV 4–6 weeks post-vaccination after which they were monitored daily for 2 weeks for the development of disease symptoms. **b** EBOV GP-specific IgG binding antibody (bAb) titers determined by IIFA at 4 weeks post-vaccination with 250 PFU (circles; YF-EBO *n* = 14; YF17D *n* = 12; sham *n* = 12) or 2500 PFU (squares; YF-EBO *n* = 5; YF17D *n* = 5). **c** ELISpot counts of IFNγ-secreting cells after EBOV GP peptide pool stimulation of isolated splenocytes from mice vaccinated with 250 PFU (circles; YF-EBO *n* = 14; sham *n* = 6) or 2500 PFU (squares; YF-EBO *n* = 5; YF17D *n* = 5). A representative image of an ELISpot well from each group is shown below the *x*-axis. **d** Percentage of IFNγ-expressing CD8^+^ cells after EBOV GP peptide pool stimulation of splenocytes from mice vaccinated with 250 PFU (YF-EBO *n* = 8; YF17D *n* = 7; sham *n* = 8). **e** Mean cumulative disease scores of mice post-challenge (YF-EBO *n* = 11; YF17D *n* = 10; sham *n* = 12), determined based on IACUC parameters including: body weight changes, body condition score, behavior and physical appearance. **f** Percentage survival post-challenge, the number of surviving mice at study endpoint are indicated within parentheses. **g** rVSV-EBOV infectious viral loads in different organs at 3 days (YF-EBO *n* = 3; YF17D *n* = 4; sham *n* = 3) and 14 days post-challenge (YF-EBO *n* = 3) quantified by virus titration on Vero E6 cells. Dashed line indicates limit of quantification (LOQ) or limit of detection (LOD). Data are median ± IQR (**b**–**d**, **g**) and two-tailed Kruskal–Wallis test was applied followed by Dunn’s multiple comparison, significant *p* values < 0.05 are indicated (**b**–**d**).

To characterize the EBOV-specific immune responses induced by YF-EBO, we vaccinated 6–8-week-old *Ifnar*^-/-^ mice^23^ with a single dose of 250 or 2500 PFU^20^ of either YF-EBO, YF17D as a matched placebo or sham as a negative control (Fig. 3a).

Four weeks post-vaccination, all YF-EBO-vaccinated mice with a single low dose of 250 PFU (*n* = 14 of 14) had seroconverted to high levels of binding antibodies (bAb) as determined by indirect immunofluorescence assay (IIFA) for EBOV GP-specific IgG (Fig. 3b). A 10-fold higher dose of 2500 PFU (*n* = 5) did not result in a significant increase in bAb titers as compared to a single low dose of 250 PFU of YF-EBO (*n* = 14; *p* > 0.9999 Kruskal–Wallis test). These bAb titers detected with our in-house IIFA significantly correlated with ELISA values^24^ of a previously validated kit^25^ (Supplementary Fig. 3a). As for other EBOV vaccines^26,27^, EBOV GP-specific neutralizing antibodies (nAbs) could not readily be detected (Supplementary Fig. 3b).

Cell mediated immune (CMI) responses were analyzed 4 weeks post-vaccination in splenocytes isolated from a subset of vaccinated mice. Splenocytes of YF-EBO-vaccinated mice with a single low dose of 250 PFU (*n* = 14) had a significantly higher number of IFNγ-secreting virus-specific T lymphocytes measured by ELISpot after stimulation with an EBOV GP peptide mix as recall antigen suggestive for successful induction of an antiviral T helper 1 (T_h_1)-polarization of CMI responses (Fig. 3c) as compared to sham (*n* = 6; *p* = 0,0014 Kruskal–Wallis test). A higher dose of 2500 PFU of YF-EBO (*n* = 5) did not result in a significant increase in IFNγ-secreting T lymphocytes as compared to a single low dose of 250 PFU of YF-EBO (*n* = 14; *p* > 0.9999 Kruskal–Wallis test). This antiviral T_h_1-polarized CMI response was further confirmed by detection of IFNγ-secreting (cytotoxic) CD8^+^ T lymphocytes by means of flow cytometry in YF-EBO vaccinated mice after EBOV GP peptide mix stimulation (Fig. 3d).

To evaluate the EBOV-specific protective efficacy induced by YF-EBO, remaining vaccinated *Ifnar*^-/-^ mice with a single low dose of 250 PFU were infected 4–6 weeks post-vaccination intraperitoneally with 100 PFU of rVSV-EBOV as established before for vigorous challenge (Fig. 3a). Most of the sham- (*n* = 11 of 12) and all YF17D-vaccinated mice (*n* = 10 of 10) rapidly developed serious disease symptoms as listed above, resulting in the development of high disease scores (Fig. 3e) and reaching humane endpoints as early as 2 days post-infection (Fig. 3f). By contrast, all YF-EBO-vaccinated mice (*n* = 11 of 11), experienced only an initial transient weight loss, associated with a moderate increase in disease scores, after which they all gained weight and remained healthy until the end of the study. A subset of mice (YF-EBO *n* = 3; YF17D *n* = 4; sham *n* = 3) were euthanized 3 days post-challenge to determine the infectious viral loads in different organs (liver, spleen, kidney, brain and lung). High infectious viral loads were detected in all the collected organs of sham- and YF17D-vaccinated mice on day 3 post-challenge (except one brain sample of a YF17D-vaccinated mouse). On the other hand, only low levels of infectious virus were detectable in the majority of the collected organs (except in the spleen) of YF-EBO-vaccinated mice on day 3 post-challenge, and no infectious virus whatsoever by the end of the study (Fig. 3g). Serum samples from a subset of mice that survived challenge were also analyzed. At 14 days post-challenge, while most of the control mice had already succumbed to challenge, all analyzed YF-EBO-vaccinated mice (*n* = 6 of 6) seroconverted to readily detectable levels of rVSV-EBOV-specific nAb titers (Supplementary Fig. 3b) and had markedly higher levels of EBOV GP-specific binding antibodies (*p* = 0,0313, Wilcoxon matched-pairs signed rank test) (Supplementary Fig. 3c) reflecting an anamnestic response following challenge.

### YFV-specific immunogenicity and protection against lethal intracranial YF17D challenge in mice

**Fig. 4:**
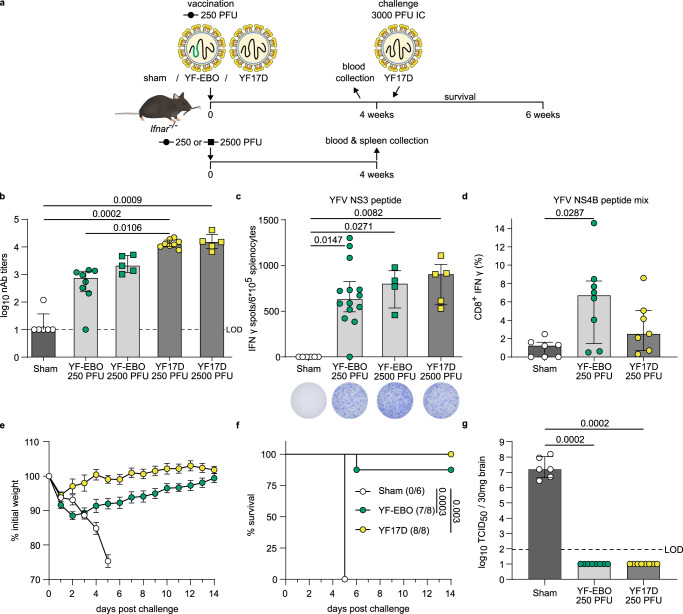
YFV-specific immunity and protection in mice. **a** *Ifnar*^-/-^ mice were vaccinated intraperitoneally with 250 (circles) or 2500 PFU (squares) of either YF-EBO, YF17D or sham. A subset of mice vaccinated with 250 PFU and all mice vaccinated with 2500 PFU were sacrificed 4 weeks post-vaccination for spleen collection. Remaining mice vaccinated with 250 PFU were challenged intracranially with 3000 PFU of YF17D after which they were monitored daily for 2 weeks for the development of disease symptoms. **b** YF17D-specific neutralizing antibody (nAb) titers at 4 weeks post-vaccination with 250 PFU (circles; YF-EBO *n* = 8; YF17D *n* = 8; sham *n* = 6) or 2500 PFU (squares; YF-EBO *n* = 5; YF17D *n* = 5). **c** ELISpot counts of IFNγ-secreting cells after YFV NS3 peptide stimulation of isolated splenocytes from *Ifnar*^-/-^ mice that were vaccinated with 250 PFU (circles; YF-EBO *n* = 14; sham *n* = 6) or 2500 PFU (squares; YF-EBO *n* = 5; YF17D *n* = 5). A representative image of an ELISpot well from each group is shown below the *x*-axis. **d** Percentage of IFNγ-expressing CD8^+^ cells after YFV NS4B peptide pool stimulation of splenocytes from mice vaccinated with 250 PFU (YF-EBO *n* = 8; YF17D *n* = 7; sham *n* = 7). One sham mice with high background staining in ICS was excluded from the analysis because it was detected as an outlier with ROUT method (*Q* = 0.1%). **e** Mean weight evolution, error bars indicate SEM (YF-EBO *n* = 8; YF17D *n* = 8; sham *n* = 6), and **f** survival curves after challenge, number of surviving mice at study endpoint are indicated within parentheses. **g** YF17D infectious viral loads in the brain at day of euthanasia determined by virus endpoint titration on BHK-21J cells. Dashed line indicates limit of detection (LOD). Data are median ± IQR and two-tailed Kruskal–Wallis test was applied followed by Dunn’s multiple comparison (**b**–**d**, **g**) and a log-rank test was applied to compare survival curves (**f**), significant *p* values < 0.05 are indicated.

To characterize the YFV-specific immunity induced by YF-EBO, we vaccinated 6–8-week-old *Ifnar*^-/-^ mice with the same single dose of 250 or 2500 PFU of YF-EBO, YF17D as a matched placebo or sham as a negative control (Fig. 4a).

Four weeks post-vaccination most of the YF-EBO-vaccinated mice with a single low dose of 250 PFU analyzed (*n* = 7 of 8) had seroconverted to YF17D-specific nAb titers (Fig. 4b) although to a somewhat lower level (2.5 log_10_-transformed geometric mean titers (GMT), 95% confidence interval (CI) 1.8–3.4) as compared to mice vaccinated with 250 PFU original YF17D (*n* = 8 of 8; 4.1 log_10_-GMT, 95% CI 4–4.2 ; *p* = 0.0260 Kruskal-Wallis-test). A higher dose of 2500 PFU of YF-EBO (*n* = 5) did not result in a significant increase in YF17D-specific nAb titers as compared to a single low dose of 250 PFU of YF-EBO (3.4 log_10_-GMT 95% CI 3–3.8, versus 2.5 log_10_-GMT, 95% CI 1.8–3.4, respectively; *p* > 0.9999 Kruskal–Wallis test).

Virus-specific CMI responses were analyzed as before in a subset of vaccinated mice 4 weeks post-vaccination, yet using YFV-derived peptides as the recall antigen. Splenocytes of YF-EBO (*n* = 14) and YF17D-vaccinated mice (*n* = 5) contained significantly higher amounts of T-cells secreting IFNγ after stimulation with a MHC-I restricted YFV NS3 peptide^28^ as compared to sham-vaccinated mice (*n* = *6; p* = 0,0147 and 0,0271, respectively, Kruskal–Wallis test). A similar level of YFV-specific antiviral T_h_1-response was observed in mice when comparing both a single low dose of 250 PFU (*n* = 14) or 2500 PFU of YF-EBO (*n* = 5) with a single dose of 2500 PFU of YF17D (*n* = 4) (Fig. 4c). This YFV-specific antiviral T_h_1-response was further confirmed by detection of IFNγ-secreting (cytotoxic) CD8^+^ T lymphocytes by means of flow cytometry in YF-EBO vaccinated mice after YFV NS4B peptide pool stimulation (Fig. 4d).

To evaluate the protective efficacy induced by YF-EBO against YFV infection, remaining vaccinated *Ifnar*^-/-^ mice were challenged with a lethal intracranial dose of 3000 PFU of YF17D (Fig. 4a). Intracranial challenge has been described previously as a stringent model to evaluate protection conferred by YF17D and YF17D-vectored vaccines in mice (ref. ^20^. and references therein). All sham-vaccinated mice (*n* = 6 of 6) developed serious disease symptoms with neurological complications such as acute weight loss, ruffled fur, hunched posture and hind limb paralysis, uniformly reaching humane endpoints as early as 5 days post-challenge. At contrary, the majority of YF-EBO- (*n* = 7 of 8) and all YF17D-vaccinated mice (*n* = 8 of 8) survived challenge, recovered rapidly, gained weight and did not develop any signs of disease (Fig. 4e, f). While initially a more pronounced weight loss was observed in YF-EBO vaccinated animals immediately after intracranial challenge (Fig. 4e), YF17D-specific nAbs were boostered in both vaccine groups to similar high, seemingly saturating levels (Supplementary Fig. 4). High infectious viral loads (Fig. 4g) at the day of euthanasia were detected in the brains of all sham-vaccinated mice (with a median infectious viral load of 10^7.2^ TCID50/30 mg brain). In contrast, no infectious virus was detected in the brains of any of the YF-EBO-vaccinated mice at the day of euthanasia; comparable to YF17D vaccination. Notably, the single YF-EBO vaccinated animal dying after intracranial challenge had detectable levels of nAb at time of infection; suggesting premature death in this case was rather linked to the harsh experimental procedure. Likewise, absence of challenge virus in the brain suggests a successful YFV immunization in all YF-EBO-vaccinated animals.

### Distinct ebolavirus vaccine candidates and humoral cross-reactivity in mice

**Fig. 5:**
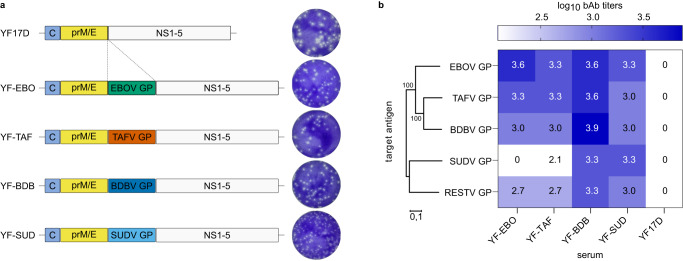
Antibody (cross)-reactivity of distinct YF17D-vectored ebolavirus vaccine candidates. **a** Schematic of YF17D-vectored ebolavirus vaccine constructs and representative images of their plaque phenotypes on BHK-21J cells, fixed 6 days post-infection. **b** Heatmap representing the different ebolavirus GPs (EBOV GP, SUDV GP, TAFV GP, BDBV GP and RESTV GP) log_10_-transformed mean binding antibody titers present in serum pools of *Ifnar*^-/-^ mice that were hyperimmunized with either YF-EBO, YF-SUD, YF-TAF, YF-BDB or YF17D determined by IIFA (*n* = 2). Phylogenetic tree based on the amino acid sequences of the different ebolavirus GPs was generated using the Neighbor-Joining method with 1000 bootstrap replications in MEGA11^59^. Small numbers at the nodes and the scale bar indicate bootstrap values and the number of amino acid substitutions per site, respectively.

Besides EBOV, Sudan virus (SUDV), Bundibugyo virus (BDBV), and Taï forest virus (TAFV) are other highly pathogenic viruses belonging to the *Ebolavirus* genus known to cause disease in humans^1^. Therefore, in addition to YF-EBO we have generated YF17D-vectored vaccine constructs expressing their respective GP, tentatively named: YF-SUD, YF-BDB and YF-TAF (Fig. 5a). Comparable to YF-EBO these vaccine constructs have a smaller plaque phenotype than parental YF17D. To evaluate ebolavirus cross-reactive humoral immune responses induced by these individual vaccine candidates, we hyperimmunized 6–8-week-old *Ifnar*^-/-^ mice repeatedly with YF-EBO, YF-SUD, YF-BDB, or YF-TAF, and YF17D as a matched negative control. The presence of specific IgG bAb against five different ebolavirus GPs (EBOV GP; SUDV GP; BDBV GP; TAFV GP; and additionally Reston virus GP, RESTV GP) was determined in pooled-sera of mice vaccinated 3-times with either vaccine candidate and represented in a heatmap as log_10_-transformed mean binding antibody titers (Fig. 5b). All vaccine candidates conferred the highest humoral immune response against their homologous target antigen. Despite the limited (55 to 73%) amino acid sequence similarity between the different ebolavirus GPs, a varying degree of cross-reactive antibodies induced by the different vaccine candidates was observed, with YF-BDB inducing cross-reactive antibodies with most pronounced breadth across heterologous ebolaviruses.

## Discussion

We describe the development, preclinical safety assessment, immunogenicity and efficacy of a dual-target YF-EBO vaccine candidate in mice. Demonstration of safety and attenuation is particularly important for live-attenuated vaccines such as YF17D-vectored vaccines. YF17D that has already been used for decades, has a favorable safety profile^29^ and is therefore recommended by the World Health Organization (WHO) for all people above 9 months of age living in YFV-endemic regions, with exception of severely immunocompromised people^30^. However, there are concerns about the very rare occurrence of serious adverse events associated with YF17D vaccination (e.g. vaccine-associated neurotropic and viscerotropic disease)^31,32^. Consistent with the moderate replicative trade-off posed by the insertion of foreign genes into the YF17D backbone, we observed that YF-EBO has an attenuated phenotype in vitro and an improved safety profile in vivo as compared to YF17D, a finding previously also corroborated for a YF17D-vectored vaccine candidate against SARS-CoV-2 following a similar vaccine design^18,33^. YF-EBO lacks the neuroinvasive properties of YF17D in immunocompromised and thus highly susceptible AG129 mice^19^ (Fig. 2b), and is attenuated compared to YF17D in a neuropotency test in neonatal BALB/c mice, while the remaining lethality induced by YF-EBO in latter model suggest sufficient potency as live-attenuated vaccine (Fig. 2c).

Genetic stability during viral growth is important for manufacturing of recombinant live-attenuated vaccines, and may become critical for YF17D-vectored vaccines especially when using larger antigens or nucleotide sequences containing complex secondary structures^34^. Nevertheless, the EBOV GP sequence showed reasonable genetic stability and remained detectable in YF-EBO for at least eight passages in vitro by both RT-PCR and immunofluorescent staining of infected cells (Supplementary Fig. 1), whereas stable antigen expression for as little as four passages has already be considered sufficient to manufacture seed lots of YF17D-vectored vaccines starting from cDNA^35^. Moreover, YF-EBO remained immunogenic and protective after inoculation of only 250 PFU which further suggests stable antigen expression during active replication of the viral vector in vivo.

A single vaccine dose as low as 250 PFU of YF-EBO induced potent EBOV GP-specific binding antibodies and EBOV-specific antiviral cellular immune response in *Ifnar*^-/-^ mice. Humoral and cellular immune responses were not markedly elevated following a 10-fold higher vaccine dose. Despite this may suggest saturation after a single dose, the propensity of live YF17D^20^ and its derivatives^36^ to trigger particularly strong adaptive immune reactions in this mouse model has to be kept in mind when designing future dose finding studies.

Nevertheless, YF-EBO induced immunity resulted in complete protection of these mice against lethal disease in a stringent surrogate EBOV infection model. Notably, the challenge model introduced and validated here uses rVSV-EBOV for vigorous challenge in immunocompromised *Ifnar*^-/-^ mice, allowing a first efficacy assessment under reduced BSL-2 containment. Furthermore, these data emphasize the improved safety of our YF17D-based vaccine in *Ifnar*^-/-^ mice (Fig. 2a) as compared to VSV-based EBOV vaccine causing lethal infection in these mice (Supplementary Fig. 2). A recent study also reported that rVSV-EBOV is neurotropic in immunocompetent neonatal mice^37^, opposite to previously reported lack of neurotropism of recombinant VSV-based vaccines such as approved Ervebo®^38^.

Immune correlates of protection for EBOV vaccines may not be universal and can vary across different vaccine platforms^39^. Yet, the presence of EBOV GP-specific binding antibodies was shown to correlate well with protection in non-human primate models across vaccine platforms^8,40^ and may also be crucial for the observed protection in YF-EBO-vaccinated mice. Consistent with other EBOV vaccines, our vaccine candidate confers protection without inducing detectable levels of EBOV GP-specific nAbs prior to challenge^26,27^.

Throughout the study we were limited to use immunocompromised *Ifnar*^-/-^ mice, since the replication of YF17D-vectored vaccines and the infection with rVSV-EBOV are highly restricted by type I IFN responses in immunocompetent wild-type mice^20,41,42^. Therefore, future mechanistic studies should address the relative contribution of humoral and cellular immune responses to the observed protection against EBOV infection in immunocompetent animal models; with extra focus on persistence of biomarkers for immunity and duration of protection.

In addition to the EBOV-specific immunogenicity, a single low dose of YF-EBO also elicited YF17D-specific nAbs and T_h_1 cellular immune responses, resulting in protection against stringent intracranial YF17D challenge in *Ifnar*^-/-^ mice^20^. Hence, YF-EBO could also be considered as a potent YFV vaccine candidate. In this way a single dual-target vaccine could help prevent the risk of new flare-ups of EVD and concomitantly contribute to initiatives such as the Eliminate Yellow Fever Epidemics (EYE) program from the WHO^43^ aiming to increase population-wide YFV coverage in endemic regions. Likewise, latent EBOV infection persisting in EVD survivors^44–47^ could be the source of new outbreaks, meaning more frequently and in absence of otherwise rare zoonotic spill-over events^48^. Such a dramatic change in the ecology of EVD asks for routine immunization in EBOV-endemic countries^10,11^ requiring safe and potent second-generation vaccines that can be readily deployed, ideally by single dosing as demonstrated here in principle for YF-EBO.

One of the concerns of viral vector vaccines is the impact of pre-existing anti-vector immunity on vaccine efficacy. However, we previously reported that pre-existing YFV-specific immunity in mice and hamsters did not reduce the efficacy of a similar YF17D-vectored SARS-CoV-2 vaccine candidate against SARS-CoV-2^49^. This suggests that it might also be feasible to use YF-EBO in YFV-seropositive populations in Africa.

Finally, we showed that YF17D as a vaccine vector is amenable to target different species from the *Ebolavirus* genus and can be used to develop filovirus vaccines that may induce cross-reactive binding antibodies. Future studies would have to address whether this observed cross-reactive humoral immunity is sufficient to also provide cross-protection against heterologous challenge; ideally in step-up models using original filoviruses under BSL4 conditions.

In conclusion, we have developed and characterized a novel dual-target single-shot YF17D-vectored EBOV vaccine candidate that provides protection against both EBOV and YFV infection in mice, which warrants further (pre-)clinical development.

## Methods

### Cells and viruses

BHK-21J (baby hamster kidney fibroblasts) cells were provided by P. Bredenbeek and maintained in minimum essential medium (MEM) (Gibco), Vero E6 (African green monkey kidney, ATCC CRL-1586) and HEK293T (human embryonic kidney cells, ATCC CRL-3216) cells were maintained in Dulbecco’s modified Eagle medium (DMEM) (Gibco). All media were supplemented with 10% fetal bovine serum (Hyclone), 2 mM L-glutamine (Gibco), 1% sodium bicarbonate (Gibco). BSR-T7/5 (T7 RNA polymerase expressing BHK-21) cells were provided by I. Goodfellow and kept in DMEM supplemented with 0.5 mg/ml geneticin (Gibco).

Replication-competent recombinant VSV-EBOV was generated by cloning the Makona EBOV GP sequence (GenBank: KY426718.1) into the multiple cloning site of pVSV-ΔG-GFP-2.6^50,51^, kindly provided by M. A. Whitt, using MluI and XmaI restriction sites. Viral recovery of rVSV-EBOV has been performed by co-transfection of BSR-T7/5 cells with 2.5 µg of pVSV-ΔG-EBOV-GP-GFP-2.6 and VSV helper plasmids encoding for VSV-N, P, L, and G proteins with a ratio of 0.87:1.43:2.25:0.5 µg of each plasmid, respectively, and amplification of the rescued virus on Vero E6 cells. This first recovery virus was plaque-purified and amplified on Vero E6 cells to generate a homogeneous virus stock of rVSV-EBOV. Sanger sequencing and immunofluorescent staining of infected cells confirmed the presence of the EBOV GP sequence in generated virus stocks. Virus titers were determined by plaque assay on Vero E6 cells, expressed as plaque forming units (PFU)/ml.

YF17D virus, Stamaril® (lot G5400) (Sanofi-Pasteur) used for intracranial challenge experiments was passaged three times in Vero E6 cells before use. Virus titers were determined by plaque assay on BHK-21J cells.

### Vaccine design and construction

Vaccine constructs were generated using an infectious cDNA clone of YF17D (in an inducible BAC expression vector pShuttle-YF17D)^36,52,53^ using standard molecular biology techniques essentially as described^18^. In brief, YF-EBO was engineered by inserting the sequence of EBOV GP strain Makona (GenBank: KM233070.1; corresponding to aa 33–676, i.e. excluding the EBOV GP signal peptide sequence; obtained after PCR on overlapping synthetic cDNA fragments; IDT) into the full-length genome of YF17D (GenBank: X03700) as translational in-frame fusion within the YF-E/NS1 intergenic region^17,54^, followed by transmembrane domains derived from WNV to ensure a proper YF topology and correct ER luminal expression of the GP antigens in the YF backbone^18^ (Fig. 1a). Infectious recombinant viruses were generated by transfection into BHK-21J cells and harvesting supernatants 3 or 4 days post-transfection when most of the cells showed signs of cytopathic effect (CPE). Clonal YF17D^55^ used as matched control vaccine was rescued similarly by transfection of pShuttle-YF17D. Infectious virus titers (PFU/ml) were determined by a plaque assay. The presence of inserted sequences in thus generated vaccine virus stocks was confirmed by Sanger sequencing. YF-SUD expressing SUDV GP (GenBank: MH121163.1), YF-TAF expressing TAFV GP (GenBank: NC_014372.1), and YF-BDB expressing BDBV GP (GenBank: KU182911.1) were generated and rescued accordingly.

### Plaque assay

Infectious virus titers of all YF17D-vectored vaccine constructs and rVSV-EBOV were determined by a plaque assay on BHK-21J or Vero E6 cells, respectively. Confluent monolayers of cells seeded in 6-well plates were infected with serial dilutions of the respective virus stock in duplicates (incubation at room temperature for YF17D and derivatives; at 37 °C for VSV derivatives). One hour post-infection cells were washed twice with PBS and overlaid with MEM containing 0.5% ultrapure low melting point agarose (Invitrogen) and incubated at 37 °C for 6 or 2 days, respectively. After which cells were fixed with 4% formaldehyde and plaques were visualized by staining with 1% crystal violet. Plaques were counted manually and resulting virus titers of each virus stock expressed as mean PFU/ml.

### Virus growth kinetics

Viral growth kinetics were determined by infection of BHK-21J cells at an MOI of 0.01. One hour post-infection, cells were washed and medium was replaced. Supernatant samples were collected every day for 5 days and frozen at −80 °C. Viral yields were determined by virus endpoint titration on BHK-21J cells.

### Immunofluorescent staining

In vitro antigen expression of the vaccine candidate was verified by immunofluorescent staining. In brief, BHK-21J cells were infected with the YF-EBO vaccine candidate. Infected cells were fixed and stained 2 days after infection. For detection of yellow fever virus antigens, polyclonal hamster anti-YF17D antiserum was used. For detection of EBOV GP antigen, mouse anti-Zaire Ebola virus GP monoclonal antibody 4F3 (0201-020; IBT Bioservices; 1:500 dilution) was used. Secondary antibodies were goat anti-mouse Alexa Fluor 594 (A-11005; Life Technologies; 1:500 dilution) and rabbit anti-hamster FITC (307-095-003; Jackson Immuno Research; 1:500 dilution). Cells were counterstained with DAPI (Sigma). All confocal fluorescent images were acquired using the same settings on a Leica TCS SP5 confocal microscope, using a HCX PL APO 63× (NA 1.2) water immersion objective.

### Analysis of genetic stability of the YF-EBO vaccine virus

To test the genetic stability of the YF-EBO vaccine virus, virus supernatants recovered from transfected BHK-21J cells (P0) were serially passaged on BHK-21J cells (P0–P10). For all passages, fresh BHK-21J cells were infected in duplicates for 1 h with a 1:10 dilution of the virus supernatant from the respective previous passage. After infection, the cells were washed twice with PBS. Supernatants of the infected cells were collected 48–72 h after infection. The presence of inserted sequences in generated passages was confirmed by RNA extraction and Dnase I treatment (Direct-zol RNA kit, Zymo Research) followed by RT–PCR (qScript XLT, Quanta) and Sanger sequencing.

To detect the co-expression of both YF17D and EBOV GP antigens in serial passages of YF-EBO, BHK-21J cells were infected (*n* = 8) with a 1:20 dilution of each serial passage (P1-P10) or YF17D as a negative control. Infected cells were fixed and stained 1 day after infection. For detection of EBOV GP antigen, three different mouse anti-Ebola virus GP monoclonal antibodies recognizing different non-overlapping epitopes: 13C6 (0201-024; IBT Bioservices; 1:1000 dilution), 4F3 (0201-020; IBT Bioservices; 1:1000 dilution) or 4G7 (MABF2111; Merck; 1:1000) were used. For detection of yellow fever virus antigens, polyclonal hamster anti-YF17D antiserum was used. Secondary antibodies were goat anti-mouse Alexa Fluor 488 (A-11001; Life Technologies; 1:500 dilution) and goat anti-hamster Alexa Fluor 647 (A-21451; Life Technologies; 1:500 dilution). After counterstaining with DAPI, fluorescence in the far-red channel (excitation at 650 nm) and the green channel (excitation at 485 nm) was measured with a Cell Insight CX5 High Content Screening platform (Thermo Fischer Scientific). All cells that showed a specific staining for YF17D antigens, characterized by a cytoplasmic enrichment in the far-red channel, were analyzed for the co-expression of EBOV GP by measuring the enrichment of cytoplasmic staining in the green channel. The percentage of YF17D-positive cells that co-expressed EBOV GP was calculated for each monoclonal antibody used.

### Animal experiments

Six-to12-week-old male and female *Ifnar*^-/-^ mice (type-I-interferon-receptor-deficient C57BL/6 mice) and 6–8-week-old male and female AG129 mice (type-I-and-II-interferon-receptor-deficient 129 mice) were used throughout this study and were bred in-house. Five-day-old BALB/c pups were purchased from Janvier Laboratories. Animals were kept in individually ventilated filtertop cages (Sealsafe Plus, Tecniplast) with a maximum of 5 mice per cage. Mice were provided with food and water ad libitum and cage enrichment (cotton and cardboard play tunnel/shelter). Mice were randomly assigned to different experimental treatment groups within one cage, to avoid any cage effect. Projects were approved by the KU Leuven ethical committee (P030-2021, P140-2016 and P100-2019), following institutional guidelines approved by the Federation of European Laboratory Animal Science Associations (FELASA). Animal health was monitored throughout the study and animals were euthanized at experimental endpoint or when pre-defined humane endpoints were reached by intraperitoneal administration of 100 µl of Dolethal® (200 mg/ml sodium pentobarbital, Vétoquinol SA, Magny-Vernois, France).

To evaluate neurovirulence and neurotropism, BALB/c mice pups and AG129^19^ mice were, respectively, intracranially or intraperitoneally inoculated with the indicated amount of PFU of YF17D or YF-EBO and monitored daily for morbidity and mortality after inoculation.

To determine rVSV-EBOV infectivity, 10–12-week-old *Ifnar*^-/-^ mice were infected intraperitoneally with either 100 or 100,000 PFU of rVSV-EBOV and monitored daily for 2 weeks for the development of disease using the Institutional Animal Care and Use Committee (IACUC) disease-scoring list (Supplementary Table 1) including following parameters: body weight changes, body-condition score, physical appearance and behavior. Mice were euthanized when they reached a total score of three or more or when they developed one of the following symptoms: paresis, paralysis or a hunched back. Organs (kidney, liver, spleen, brain and lung) were collected at the day of euthanasia for endpoint virus titrations.

Six-to-8-week-old *Ifnar*^-/-^ mice were intraperitoneally vaccinated with 250 or 2500 PFU of YF-EBO as indicated. As a control, two groups were vaccinated with either YF17D^23^ or sham (MEM with 2% FBS). Of note, the intraperitoneal route of immunization for YF17D and derivatives thereof in small animal models has been intensively validated by us before^18,20,49,56^ and successfully bridged to the clinically relevant subcutaneous route in a non-human primate model^18^. All mice were bled 4 weeks post-vaccination for indirect immunofluorescence assays (IIFA) and serum neutralization tests (SNT). A subset of mice was euthanized at 4 weeks post-vaccination and spleens were collected for ELISpot cytokine detection or flow cytometry analysis. For rVSV-EBOV challenge, mice were intraperitoneally infected with a lethal dose of 100 PFU of rVSV-EBOV 4–6 weeks post-vaccination. Mice were monitored daily for 2 weeks for the development of disease using the IACUC disease-scoring list (Supplementary Table S1) and were euthanized when humane endpoints were reached, as described above. A subset of mice was euthanized at 3 days post-challenge and organs (kidney, liver, spleen, brain, and lung) were collected for endpoint virus titration. For YFV challenge, mice were inoculated with a lethal dose of YF17D via the intracranial route. Four weeks after intraperitoneal vaccination with 250 PFU of YF-EBO, YF17D, or sham, mice were anesthetized by intraperitoneal injection of a mixture of xylazine (16 mg/kg, XYL-M, V.M.D.), ketamine (40 mg/kg, Nimatek, EuroVet) and atropine (0.2 mg/kg, Sterop). After confirmed anesthesia, mice were inoculated intracranially with 30 μl containing 3000 PFU of YF17D, and then monitored daily for signs of disease and weight change for 2 weeks. Mice were euthanized on the basis of morbidity (paralysis, paresis, hunched posture and ruffled fur) or weight loss of more than 20% or a quick weight loss of more than 10% in 1–2 days. Brains were collected at the day of euthanasia for endpoint virus titration.

Hyperimmune serum was generated by vaccinating *Ifnar*^-/-^ mice three times intraperitoneally in 3 consecutive weeks with the respective vaccine candidate (YF-EBO, YF-TAF, YF-BDB, YF-SUD, or YF17D). Serum was collected 5 weeks after the first vaccination and pooled for each individual vaccine candidate for analysis in an IIFA for the presence of binding antibodies against distinct ebolavirus GPs.

### Detection of total binding IgG by indirect immunofluorescence assay (IIFA)

To detect EBOV-specific binding antibodies in mouse serum, an in-house-developed IIFA was used. HEK293T cells seeded in 96-well plates were transiently transfected with either pCMV-EBOV-GP-IRES-RFP or pCMV-RABV-CVS-G-IRES-RFP causing overexpression of the target antigen (EBOV GP) and RFP or an irrelevant glycoprotein (RABV-CVS-G) and RFP, respectively. To determine EBOV GP binding antibody end titers, 1/2 serial serum dilutions were made in parallel on EBOV GP-expressing cells and RABV-CVS-G expressing cells. Goat-anti-mouse IgG Alexa Fluor 647 (A-21236; Life Technologies; 1:500 dilution) were used as secondary antibody. After counterstaining with DAPI, fluorescence in the red channel (excitation at 560 nm) and the far-red channel (excitation at 650 nm) was measured with a Cell Insight CX5 High Content Screening platform (Thermo Fischer Scientific). Specific EBOV GP staining is characterized by cytoplasmic (endoplasmic reticulum) enrichment in the far-red channel. To quantify this specific EBOV GP staining, the difference in cytoplasmic and nuclear signal for the RABV-CVS-G expressing cells was subtracted from the difference in cytoplasmic and nuclear signal for the EBOV GP expressing cells. All positive values were considered as specific EBOV GP staining. The IIFA end titer of a sample is defined as the highest dilution that scored positive in this way. Cross-reactive binding antibodies against distinct ebolavirus GPs in hyperimmune serum were determined in a similar way with an IIFA on transfected cells that expressed the respective target antigen (EBOV GP, TAFV GP, BDBV GP, SUDV GP or RESTV GP) and RFP, the read-out was done on a DMi8 microscope (Leica) with fixed microscope settings.

### Enzyme-linked immunosorbent assay (ELISA)

To validate our in-house developed IIFA assay to detect EBOV-specific binding antibodies, we used a mouse anti-ZEBOV-GP IgG ELISA kit (AE-320600-1, Alpha Diagnostic) according to manufacturer instructions. Briefly, EBOV-GP specific IgG antibodies were measured in duplicates by stepwise addition of the serially diluted serum samples to the ZEBOV-GP coated ELISA plate, a horseradish peroxidase conjugated detection antibody and tetramethylbenzidine substrate, reaction was stopped after 10 min by addition of diluted sulfuric acid. Absorbance was measured at 450 nm and background at 630 nm was subtracted.

### YFV seroneutralization test

YF17D-specific neutralizing antibodies were determined using a fluorescence-based seroneutralization assay that had been developed and validated in house and employs a mCherry-tagged variant of YF17D virus^57^. In brief, serum dilutions were incubated in 96-well plates with the YF17D–mCherry virus for 1 h at 37 °C, after which serum–virus complexes were transferred for 72 h to BHK-21J cells. The percentage of mCherry-expressing cells was quantified on a Cell Insight CX5/7 High Content Screening platform (Thermo Fischer Scientific) and half-maximal inhibitory serum neutralization titers (SNT_50_) were determined by fitting the serum neutralization dilution curve that is normalized to a virus (100%) and cell control (0%) in Graphpad Prism (GraphPad Software).

### rVSV-EBOV seroneutralization test

To quantify EBOV neutralizing antibodies, serum samples were serially diluted and incubated for 1 h at 37 °C with 1000 PFU of rVSV-EBOV, which contains an additional transcription unit encoding for GFP, and inoculated overnight on Vero E6 cells. The percentage of GFP expressing cells was quantified on a Cell Insight CX5 High Content Screening platform (Thermo Fischer Scientific) and half-maximal inhibitory SNT_50_ values were determined^58^ by fitting the serum neutralization dilution curve that is normalized to a virus (100%) and cell control (0%) in Graphpad Prism (GraphPad Software).

### ELISpot

Collected spleens were passed through a cell-strainer (70 μm) to obtain single-cell suspensions and red blood cells were lysed using RBC lysis-buffer (eBioscience). ELISpot assays for the detection of IFNγ-secreting mouse splenocytes were performed with mouse IFNγ kit (ImmunoSpot MIFNG-1M/5, CTL Europe) according to manufacturer instructions. Briefly, 6×10^5^ Splenocytes were stimulated with either an EBOV GP peptide pool (PepMix™ Zaire Ebola (GP/Kikwit-95), 0.25 µM/peptide, JPT peptide technologies), a YF17D NS3 peptide (ATLTYRML, NS3_268–275_, 5 μM, Eurogentec, Seraing, Belgium) or an ovalbumin peptide (SIINFEKL, OVA_257–264_, 5 μM, Eurogentec, Seraing, Belgium). After 48 h of incubation at 37 °C, IFNγ spots were visualized by stepwise addition of a biotinylated detection antibody, a streptavidin-enzyme conjugate and the substrate. Spots were counted using an ImmunoSpot S6 Universal Reader (CTL Europe) and normalized by subtracting spots numbers from control samples (incubated with irrelevant ovalbumin peptide) from the spot numbers of corresponding stimulated samples. Negative values were corrected to zero.

### ICS and flow cytometry

Fresh mouse splenocytes were stimulated with 1.2 µM/peptide of EBOV GP peptide pool (PepMix™ Zaire Ebola (GP/Kikwit-95) JPT peptide technologies), or 1.5 µM/peptide YF17D NS4B peptide pool (PepMix™ Yellow fever (NS4B) JPT peptide technologies) or with medium supplemented with DMSO. After 48 h of incubation at 37 °C, cells were treated with 1× Brefeldin A (ThermoFisher Scientific) for 4 h at 37 °C, thoroughly washed and stained with Zombie Aqua (Zombie Aqua™ Fixable Viability Kit, Biolegend) and FcgR block (0.5 µl/well, FcR Blocking Reagent mouse, Miltenyi Biotec) for 15 min at RT in the dark. Cells were washed again and were stained with surface markers BUV395 anti-CD3 (clone 17A2; 740268, ThermoFisher Scientific, 1:167 dilution), BV785 anti-CD4 (clone GK1.5; 100453; Biolegend; 1:100 dilution), and APC/Cyanine7 anti-CD8 (clone 53-6.7; 100714, Biolegend, 1:100 dilution) in Brilliant Staining Buffer (BD Biosciences) and incubated for 25 min on ice in the dark. The cells were thoroughly washed again followed by fixation and permeabilization **(**eBioscience™ Foxp3/Transcription Factor Staining Buffer Set, Invitrogen) for 30 min on ice in the dark. Subsequently the cells were washed with permeabilization buffer (eBioscience™ Foxp3 / Transcription Factor Staining Buffer Set, Invitrogen) and stained for 45 min on ice in the dark with an APC anti-IFNγ (clone XMG1.2; 505810; Biolegend; 1:100 dilution) intracellular marker. After a final washing step, analysis was performed with a BD LSRFortessa X20 device. Data was gated using FlowJo software 10.8.1 (BD) (strategy shown in Supplementary Fig. 5) and all samples were normalized by subtraction of peptide-stimulated cells by background from non-stimulated cells of the same animal.

### Endpoint virus titrations

To quantify infectious rVSV-EBOV or YF17D viral loads in the organs of challenged mice, endpoint titrations were performed on confluent Vero E6 cells or BHK-21J cells, respectively. A piece of each collected organ (kidney, liver, spleen, brain, and lung) weighing approximately 30 mg was homogenized using bead disruption (Precellys) in 350 µl MEM and centrifuged (10,000 × *g*,10 min, 4 °C) to pellet cell debris. Viral loads were calculated by the Reed and Muench method and expressed as TCID50 per 30 mg tissue.

### Data analysis

All statistical analyses were performed using GraphPad Prism 9 software (GraphPad, San Diego, CA, USA). Results are presented as medians ± IQR or mean ± SEM as indicated. Statistical differences for multiple comparisons were analyzed using Kruskal–Wallis with Dunn’s multiple comparisons test, survival curves were analyzed using a log-rank test (Mantel–Cox), repeated measures were analyzed using a Wilcoxon matched-pairs signed rank test and all tests were considered statistically significant at *p* values < 0.05.

## Supplementary information

Supplemental Material

## Data Availability

All data supporting the findings in this study are available from the corresponding author upon request.
