# Supplementary material for: YF17D-vectored Ebola vaccine candidate protects mice against lethal surrogate Ebola and yellow fever virus challenge
Source: NPJ Vaccines. 2023 Jul 11;8:99. doi: 10.1038/s41541-023-00699-7 (PMC10336040; doi:10.1038/s41541-023-00699-7)
Supplement: Supplementary file 1 — Supplemental Material [file 41541_2023_699_MOESM1_ESM.pdf]

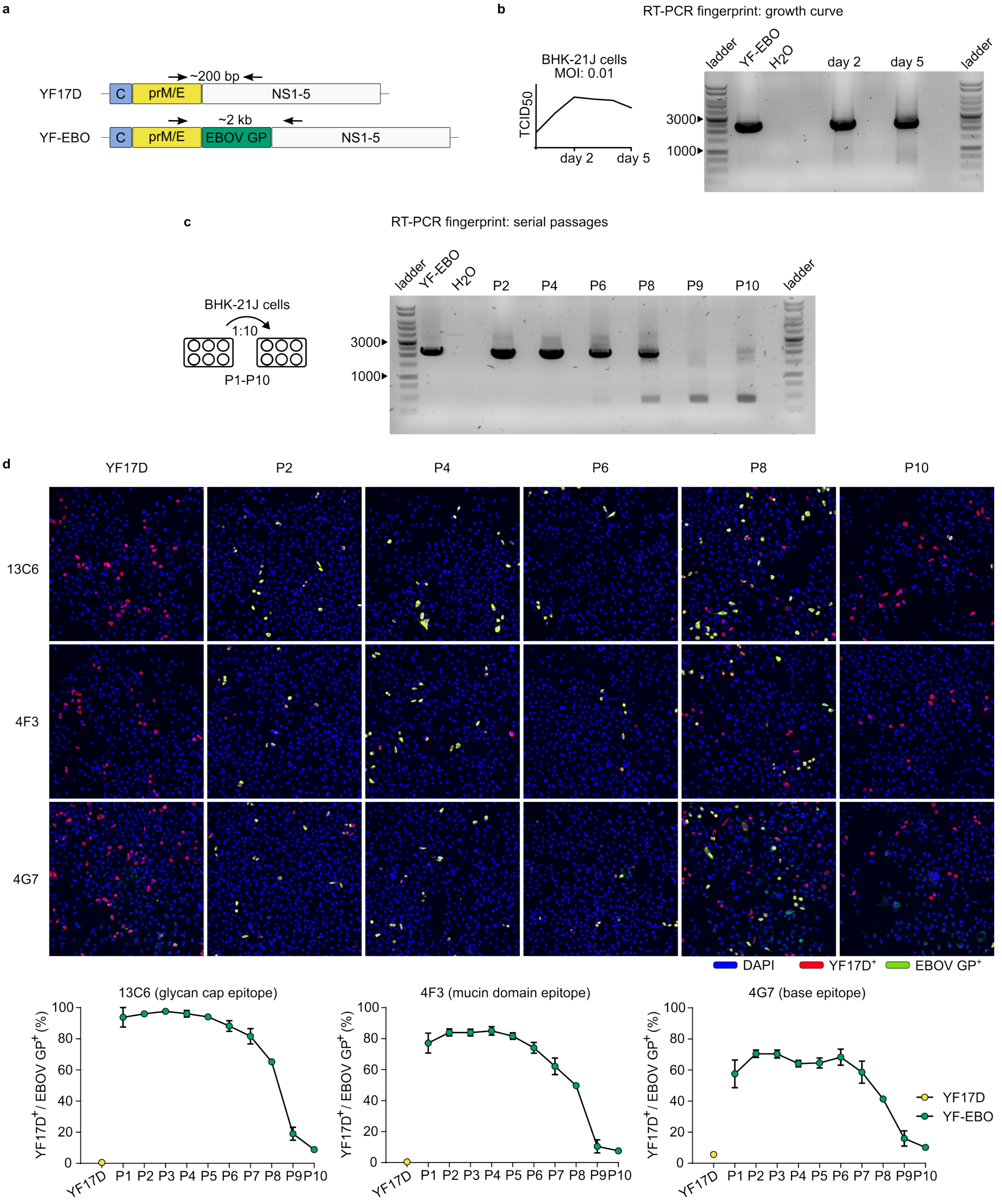

Supplementary Figure 1: Genetic stability of YF-EBO

Related to figure 1. **a.** Schematic representation of RT-PCR based detection of the EBOV GP antigen. Arrows indicate primer binding sites on the viral genome. **b.** RT-PCR fingerprint of viral RNA extracted from supernatants of day 2 and day 5 of growth curve (Fig. 1c) and **c.** viral RNA extracted from infected BHK-21J supernatants of serially passaged YF-EBO (P2–P10). PCR amplicons of pShuttle-YF-EBO were amplified using the same primer pair and served as positive control. H_2O was included as a negative control. ladder, 1-kb DNA ladder. **d.**

Representative overlay images (10x objective) of immunofluorescent stainings which detect the co-expression of both YF17D (red) and EBOV GP (green) antigens and nuclei (stained with DAPI, blue) in BHK-21J cells infected with serial passages of YF-EBO (P1-10). Three different monoclonal antibodies targeting different epitopes of EBOV GP (13C6, 4F3 and 4G7) were used in the stainings. Graphs show the mean percentage of YF17D-infected cells that co-express EBOV GP quantified by high content image analysis. Cells infected with YF17D served as a negative control. Error bars indicate SEM (*n* = 8).

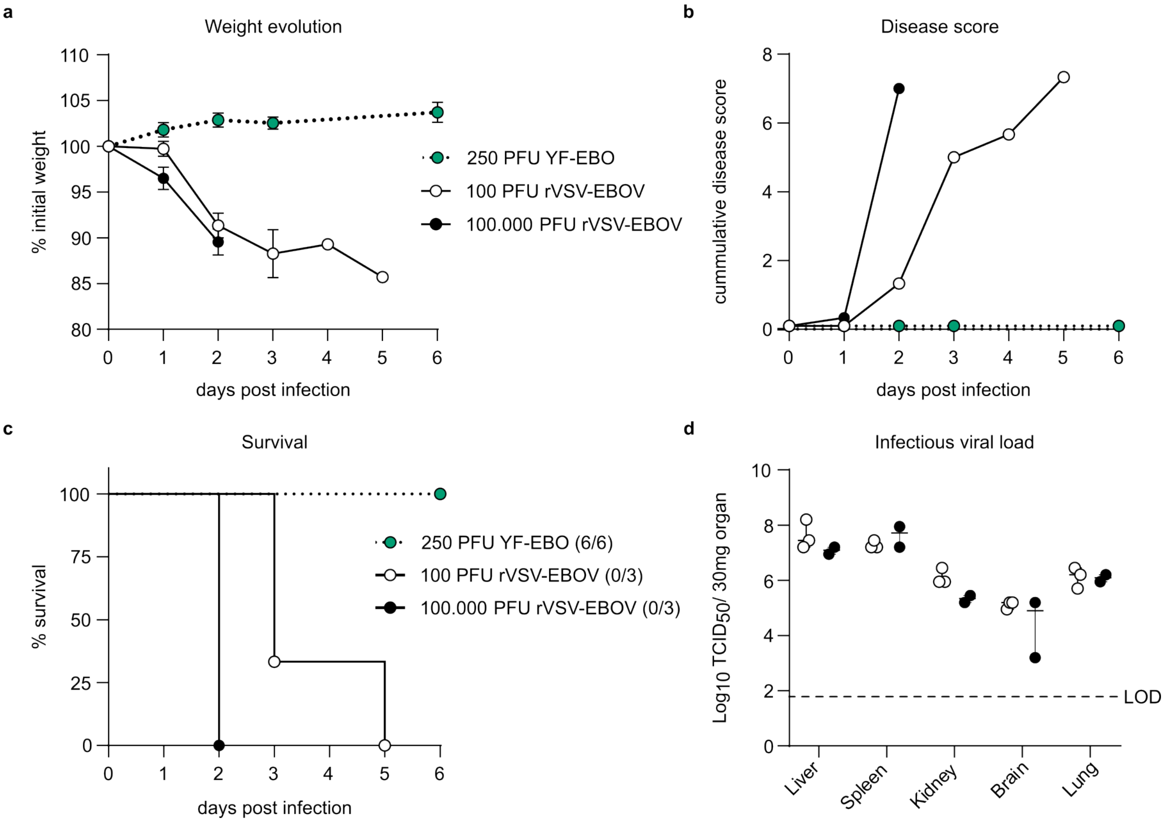

Supplementary Figure 2: rVSV-EBOV infection in mice

Ifnar $^{-/-}$ mice were infected intraperitoneally with 100 (n = 3) or 100.000 PFU (n = 3) of rVSV-EBOV and monitored for the development of disease symptoms. **a.** Weight evolution after infection with rVSV-EBOV, as a comparison the dotted line represents weight evolution of *Ifnar*$^{-/-}$ mice (n = 6) after vaccination (intraperitoneal inoculation with 250 PFU of YF-EBO; data as in Figure 2a). Error bars represent SEM. **b.** Mean cumulative disease score, based on IACUC parameters (see Supplementary table S1) including: body weight changes, body condition score, behaviour and physical appearance. **c.** Survival curve. The number of surviving mice at study endpoint are indicated within parentheses. **d.** rVSV-EBOV infectious viral loads in different organs collected at the day of euthanasia and quantified by virus titration on Vero E6 cells. Data are median ±IQR, dashed line represents limit of detection (LOD) (d)**.**

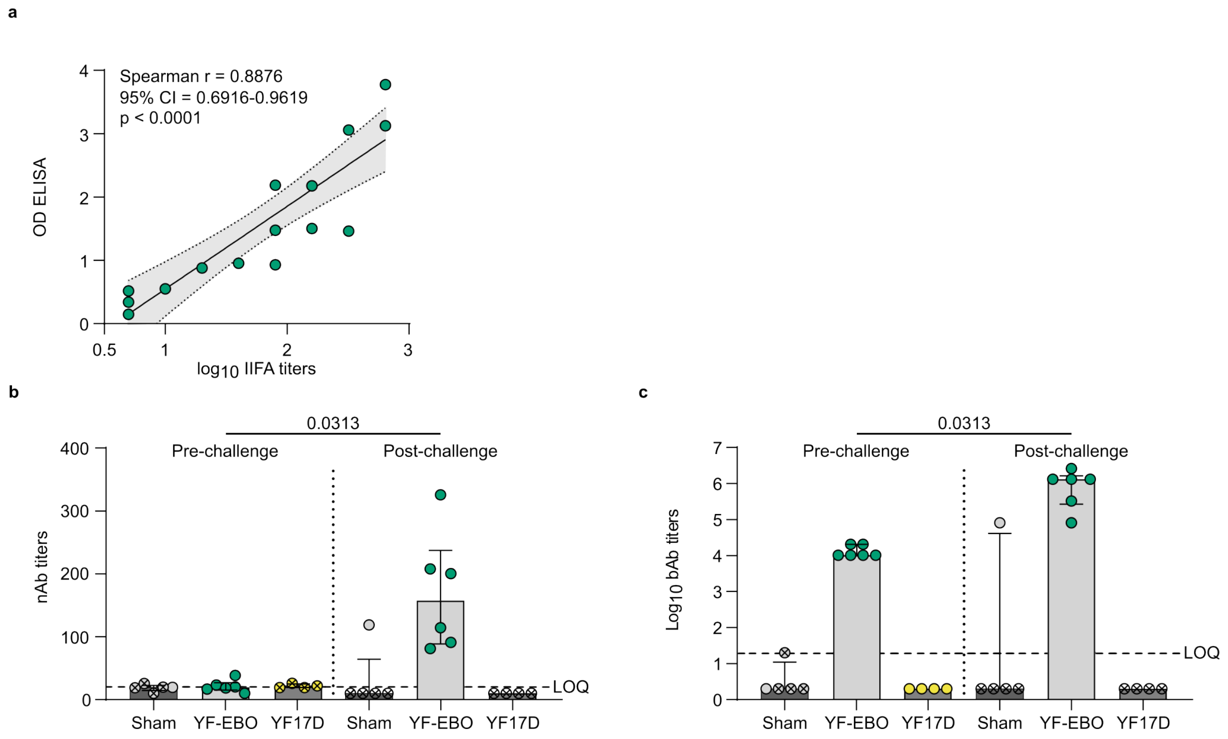

Supplementary Figure 3: Correlation between EBOV GP specific IIFA and ELISA and comparison of EBOV-specific humoral immunity pre- and post-challenge

Related to figure 3. **a.** EBOV GP IgG binding antibody (bAb) titers were determined in serially diluted serum samples (n=16) from vaccinated mice (n=3) by IIFA and ELISA. Linear regression analysis was performed to calculate the Spearman correlation coefficient between IIFA titers and ELISA optical densities (OD) and is indicated by a black solid line, 95% confidence intervals (CI) are indicated by grey shaded areas. The Spearman correlation coefficient r, 95% CI and p-value are indicated. **b.** Pre-challenge (four weeks post-vaccination) and post-challenge (2 weeks post-challenge) rVSV-EBOV GP-specific neutralizing antibody (nAb) titers determined by rVSV-EBOV seroneutralization test and **c.** EBOV GP-specific IgG binding antibody (bAb) titers determined by IIFA, present in serum of mice vaccinated with 250 PFU (YF-EBO n = 6; YF17D n = 4; sham n = 5). Mice that succumbed to rVSV-EBOV infection are represented with an ‘x’. Dashed line represents limit of quantification (LOQ), data are median ±IQR and two-tailed Wilcoxon matched-pairs signed rank test was applied, significant p-values < 0.05 are indicated (b-c).

a

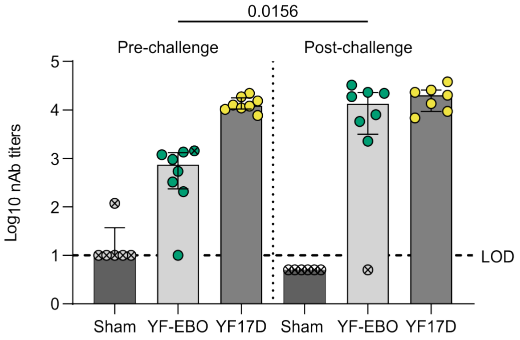

Supplementary Figure 4: Comparison of YFV-specific humoral immunity pre- and post-challenge

Related to figure 4. **a.** Pre-challenge (four weeks post-vaccination) and post-challenge (2 weeks post-challenge) YF17D-specific neutralizing antibody (nAb) titers determined by YFV seroneutralization test, present in serum of mice vaccinated with 250 PFU (YF-EBO n = 8; DYF17D n = 8; sham n = 6). Mice that succumbed to intracranial YF17D infection are represented with an 'x'. Dashed line represents limit of detection (LOD), data are median ±IQR and two-tailed Wilcoxon matched-pairs signed rank test was applied, significant p-values < 0.05 are indicated (a).

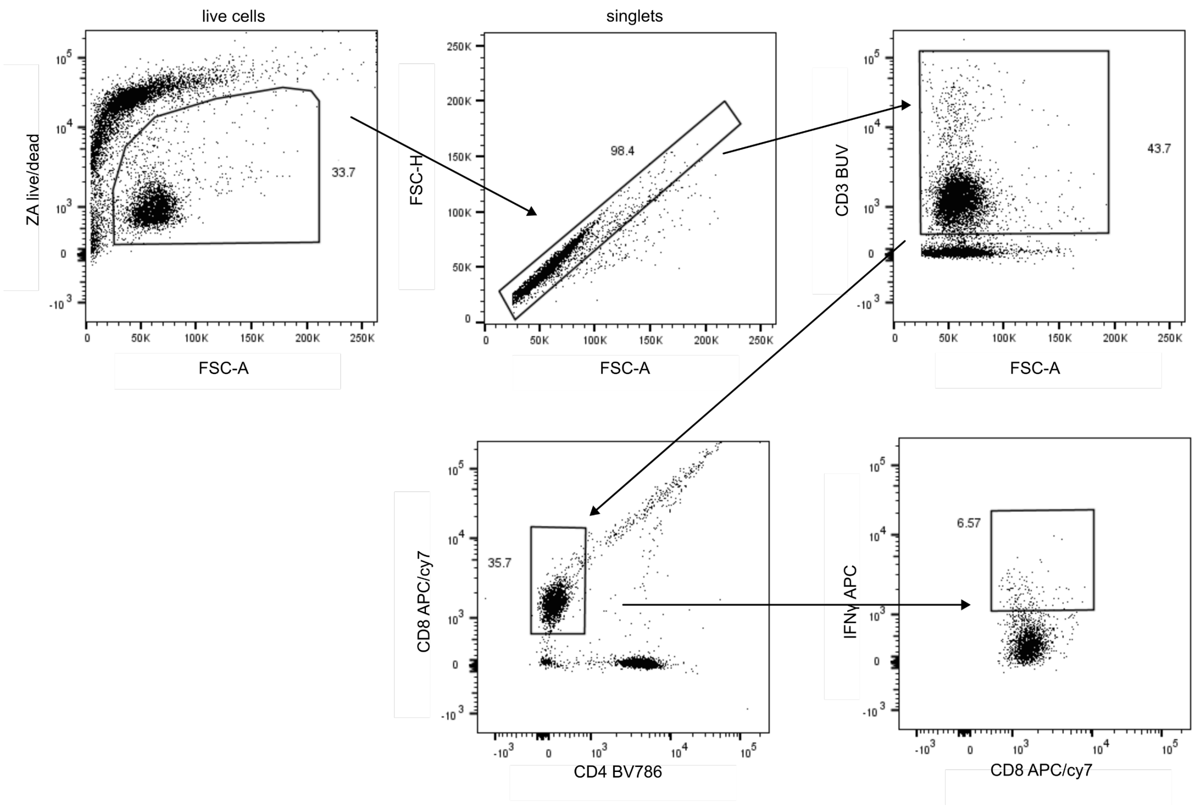

Supplementary Figure 5: Gating strategy

First, live cells were selected by gating out Zombie Aqua (ZA)-positive and low forward scatter (FSC) events. Then, doublets were eliminated in an FSC-H versus FSC-A plot. T cells (CD3+) were stratified into CD8 T cells (CD8+). Boundaries defining positive and negative populations for intracellular marker (IFNγ) were set on the basis of non-stimulated control samples.

Uncropped gel images: supplementary figure 1

Panel b

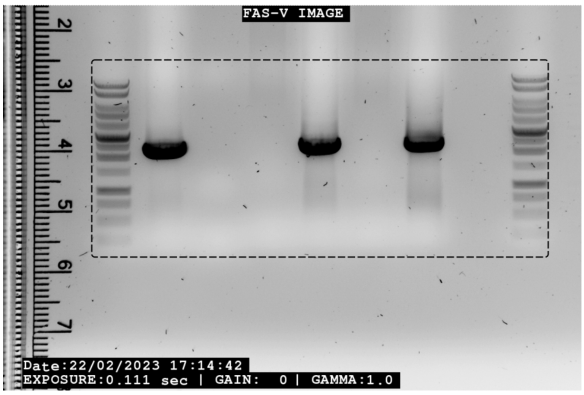

Panel c

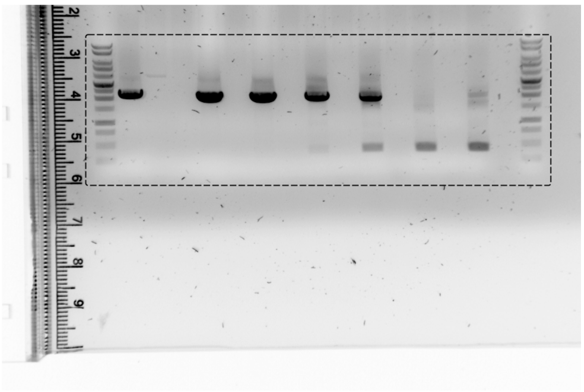

Supplementary Figure 6: Uncropped gels

Uncropped RT-PCR gels. Dotted lines specify each area which was cropped to generate respective panels in supplementary Fig.1b and 1c as indicated.

Supplementary Table 1: IACUC disease-scoring list

Category	Score	Criteria
Body weight changes	0	Normal
	1	<10% Weight loss
	2	10-15% Weight loss
	3	>20% Weight loss
Body-condition score	0	Well-conditioned (vertebrae, pelvic, or spinal bones not prominent)
	1	Under-conditioned (evident vertebral segmentation; pelvic bones readily palpable)
	2	Emaciation (skeletal structures extremely prominent; little or no flesh cover)
Physical appearance	0	Normal
	1	Lack of grooming
	2	Rough coat, nasal/ocular discharge
	3	Very rough coat, abnormal posture
Behaviour	0	Normal
	1	Minor changes (e.g., limping)
	2	Abnormal; reduced mobility, inactive
	3	Unsolicited vocalizations, self-mutilation, either very restless or immobile
